# MALDI-TOF Identification of the Human Gut Microbiome in People with and without Diarrhea in Senegal

**DOI:** 10.1371/journal.pone.0087419

**Published:** 2014-05-01

**Authors:** Bissoume Samb-Ba, Catherine Mazenot, Amy Gassama-Sow, Grégory Dubourg, Hervé Richet, Perrine Hugon, Jean-Christophe Lagier, Didier Raoult, Florence Fenollar

**Affiliations:** 1 Unité de Recherche sur les Maladies Infectieuses et Tropicales Emergentes (URMITE) UM63, CNRS 7278, IRD 198, INSERM 1095, Aix-Marseille Université, Marseille, France and Dakar, Senegal; 2 Unité de Bactériologie Expérimentale, Institut Pasteur de Dakar, Dakar, Senegal; Indian Institute of Science, India

## Abstract

**Background:**

In Africa, there are several problems with the specific identification of bacteria. Recently, MALDI-TOF mass spectrometry has become a powerful tool for the routine microbial identification in many clinical laboratories.

**Methodology/Principal Findings:**

This study was conducted using feces from 347 individuals (162 with diarrhea and 185 without diarrhea) sampled in health centers in Dakar, Senegal. Feces were transported from Dakar to Marseille, France, where they were cultured using different culture conditions. The isolated colonies were identified using MALDI-TOF. If a colony was unidentified, 16S rRNA sequencing was performed. Overall, 2,753 isolates were tested, allowing for the identification of 189 bacteria from 5 phyla, including 2 previously unknown species, 11 species not previously reported in the human gut, 10 species not previously reported in humans, and 3 fungi. 2,718 bacterial isolates (98.8%) out of 2,750 yielded an accurate identification using mass spectrometry, as did the 3 *Candida albicans* isolates. Thirty-two bacterial isolates not identified by MALDI-TOF (1.2%) were identified by sequencing, allowing for the identification of 2 new species. The number of bacterial species per fecal sample was significantly higher among patients without diarrhea (8.6±3) than in those with diarrhea (7.3±3.4; *P* = 0.0003). A modification of the gut microbiota was observed between the two groups. In individuals with diarrhea, major commensal bacterial species such as *E. coli* were significantly decreased (85% versus 64%), as were several *Enterococcus* spp. (*E. faecium* and *E. casseliflavus*) and anaerobes, such as *Bacteroides* spp. (*B. uniformis* and *B. vulgatus*) and *Clostridium* spp. (*C. bifermentans*, *C. orbiscindens*, *C. perfringens*, and *C. symbosium*). Conversely, several *Bacillus* spp. (*B. licheniformis*, *B. mojavensis*, and *B. pumilus*) were significantly more frequent among patients with diarrhea.

**Conclusions/Significance:**

MALDI-TOF is a potentially powerful tool for routine bacterial identification in Africa, allowing for a quick identification of bacterial species.

## Introduction

There are several problems in the specific identification of bacterial infections in Africa. Currently, bacterial identification is based on phenotypic tests, including Gram staining, bacterial culture, culture growth characteristics, and biochemical profiles. Even if culture processes are available in major hospitals in Africa, there are limitations to the performance of biochemical identification methods. Such traditional methods require the possession of many API strips including API-20E, API-20NE, API Staph kits, and API Anaerobe kits and many unique reagents that should be stocked under specific conditions and have expiration dates. Biochemical methods are time consuming. They often required knowledge about the type of microorganism being tested, and fail to accurately identify several bacteria species [1], [2].

Five years ago, a revolution occurred in bacteriology with the advent of the routine identification of bacteria by matrix-assisted laser desorption ionization time-of-flight mass spectrometry (MALDI-TOF) [1], [3]–[5]. Currently, this technique allows accurate identification of bacteria without a priori knowledge of the type of microorganism. This technique is in widespread use in many clinical laboratories in Europe [1], [3], [4], [6], [7]. This method allows for the detection of bacteria in less than 1 hour and is cost effective. Thus, this technique has become a powerful tool for routine identification and could replace Gram staining and biochemical identification, but to this point, many studies using this technique have been mainly performed in Europe [2].

The bacterial repertoire is different depending on the environment from which the microorganisms are obtained [8], [9]. For example, differences at the species level have been observed among the microbes in the human between Asian versus American people and European versus African people [10], [11]. Another recently developed high-throughput method involves the combination of culturomics using a large panel of media incubated at several atmospheric conditions and MALDI-TOF mass spectrometry for the quick and accurate identification of a large number of colonies [12]–[14].

In this study, we evaluated the effectiveness of MALDI-TOF mass spectrometry on the identification of bacterial species isolated from feces from Senegalese patients with and without diarrhea by combining several culture conditions and rapid mass spectrometry identification.

## Materials and Methods

### Ethics Statement

All aspects of this study were approved by the National Ethical Committee (CNERS) of Senegal (SEN25/07). Written consent was obtained for all participants. For children, their parents or guardians provided also a written informed consent.

### Patient Recruitment and Sample Management

This study was based on 347 individuals, adults and children, sampled from March 2009 to January 2010∶162 individuals with diarrhea and 185 without diarrhea (Table 1). Five health centers in Dakar, Senegal and its suburbs (Dominique-Pikine, Sicap Mbao, Roi Baudoin, Institut d’Hygiène Sociale, and Saint Martin) were included. Stool samples were collected from children and adults who attended these health centers. Control patients were hospitalized patients or outpatients without intestinal pathogens or recent treatment with antibiotics.

**Table 1:** Population description.

	Patients		Controls	
Age (years)	Number	%	Number	%
[0–5]	71	43.8	9	4.9
[5]–[20]	35	21.6	46	24.9
[20	56	34.6	130	70.2
**Total**	**162**	**100.00**	**185**	**100.00**

Stool specimens were collected in special sterile stool containers or with swabs for stool samples collected from infants. All stool samples were labeled and transported in cool boxes for examination within 24 hours of collection to Institut Pasteur de Dakar (Senegal). At the laboratory, macroscopic and microscopic analyses were performed on fresh stool samples to look for enteric pathogens including eggs, cysts, and trophozoites of intestinal parasites as well as enteric viruses. Stool samples were preserved in two Nunc tubes (Fisher Thermo Scientific, Denmark) and stored at −20°C. They were transported from Dakar to Marseille, France in ice packs.

### Culturomics Methods

To enumerate the number of colony forming units (CFU) in the stool samples, 1 g of pasty stool was diluted in 9 ml of phosphate buffered saline (PBS), and 100 µl of watery stool was diluted in 900 µl of PBS. The diluted samples were introduced with a syringe for preincubation into aerobic and anaerobic blood culture bottles (BD Bactec Plus Lytic/10 Anaerobic, Aerobic, 39 Heidelberg, Germany) for 24 hours before being inoculated on agar plates as it has been previously reported that this strategy allowed the growth of bacterial species, mainly anaerobic, that were not detected by standard axenic culture, species [12], [15]. Plates for anaerobic culture were pre-incubated for 24 h anaerobically. To identify the maximum number of bacterial species, stool samples were diluted from 10^−1^ to 10^−10^ and inoculated on agar plates using nine different culture conditions that had been previously determined to be the most useful (Table 2) [12]. The microaerophilic and anaerobic incubations were carried out using microaerophilic bags (Oxoid, Basingstoke, England), anaerobic jars (Mitsubishi) and atmosphere generators (BD Diagnostics, Heidelberg, Germany). Each agar plate was carefully observed after 2 and 7 days of incubation. Any isolated colony was applied to mass spectrometry for identification.

**Table 2:** Culture media and conditionings used in this study.

Media	Culture conditions	Suppliers
**Direct inoculation**		
5% sheep blood agar	Aerobe, 37°C, 48 hours	Biomérieux, Marcy l’Etoile, France
5% sheep blood agar	Anaerobe, 37°C, 48 hours	Biomérieux
MacConkey	Aerobe, 37°C, 48 hours	Biomérieux
BCYE	Aerobe with 2.5% CO_2_, 37°C, 5 days	Biomérieux
BCP	Aerobe, 37°C, 48 hours	Biomérieux
LAMVAB	Anaerobe, 37°C	Home-made*
**Inoculation in a blood culture bottle for 24 h, followed by inoculation in**		
Columbia	Aerobe, 37°C, 3 days	Biomérieux
MacConkey	Aerobe, 37°C, 1 day	Biomérieux
Columbia	Anaerobe, 37°C, 3 days	Biomérieux

BCYE: Buffered Charcoal Yeast Extract; BCP: Bromocresol Purple; LAMVAB: Lactobacillus Anaerobic MRS with Vancomycin and Bromocresol green. *from Hartemink *et al*. [15].

### Identification Using Mass Spectrometry

The isolated colonies were deposited on a MALDI-TOF target microflex (Bruker Daltonik, Wissembourg, France) and overlaid with matrix solution, a saturated solution of α- cyano-4-hydroxycinnamic acid in 50% acetonitrile and 2.5% trifluoroacetic acid, after air-drying at room temperature for 5 minutes. Each colony was picked from an Eppendorf tube containing the Trypticase-Casein-Soy (AES) culture medium stored at 37°C. Broth culture-specific thioglycollate (BD Diagnostics) was used for anaerobes. Two spots were examined for each colony. Each deposit was covered with 2 µl of the matrix solution. The Biotyper software was used to compare the protein profile of the bacteria obtained from a database (Bruker and the base of the Timone hospital) of protein profiles regularly updated based on the results of clinical diagnosis. This software takes into account a maximum of 100 mass peaks between 3,000 and 15,000 Da. A score >1.9 indicates a high-level identification of genus and species. A score >1.7 indicates the identification of genus but not species, and a score lower than 1.7 indicates no identification of bacteria. If the species was still not accurately identified by MALDI-TOF after two attempts, the isolate was analyzed by 16S rRNA sequencing.

### 16S rRNA Amplification and Sequencing Identification

Bacterial DNA was extracted using the MagNA Pure LC kit DNA isolation kit III (Roche, France) with the MagNA Pure LC instrument, according to the manufacturer’s instructions. The 16S rRNA gene was amplified by PCR using the universal primer pair *fd1* and *rp2* and an annealing temperature of 52°C, as described elsewhere [16]. PCR products were purified using the PCR kit Nucleofast 96 (Macherey-Nagel, Hoerdt, France). Sequencing reactions were performed with the sequencing kit Big Dye Terminator version 1.1 (Perkin-Elmer, Coignieres, France) with primers *536F*, *536R, 800F*, *800R*, *1050F*, and *1050R* (Table 3). Products of the sequencing reactions were purified and the sequences analyzed on an ABI PRISM 3130X Genetic Analyzer (Applied Biosystems, California, USA). The obtained sequences were compared with the GenBank database using BLAST software. A threshold value of similarity ≥98.7% was used for identification at the species level. Below this value, sequences were repeated to confirm the first obtained results. A new species was suspected when the similarity in the GenBank database with described bacteria was <98.7% [17], [18].

**Table 3:** Primers used for 16S rRNA PCR and sequencing.

Primers	Sequences (5′–3′)	Annealing temperature
FD1	AGAGTTTGATCCTGGCTCAG	52°C
RP2	ACGGCTACCTTGTTACGACTT	52°C
536F	CAGCAGCCGCGGTAATAC	50°C
536R	GTATTACCGCGGCTGCTG	50°C
800F	ATTAGATACCCTGGTAG	50°C
800R	CTACCAGGGTATCTAAT	50°C
1050F	TGTCGTCAGCTCGTG	50°C
1050R	CACGAGCTGACGACA	50°C

### Statistical Analyses

Statistical analyses were performed using EpiInfo6 software (http://www.cdc.gov/epiinfo/Epi6/EI6dnjp.htm). The results were concluded to be statistically significant when *P*<0.05. The corrected chi-squared test or Fisher’s exact test was used where indicated.

## Results

### Culture

**Figure 1:**
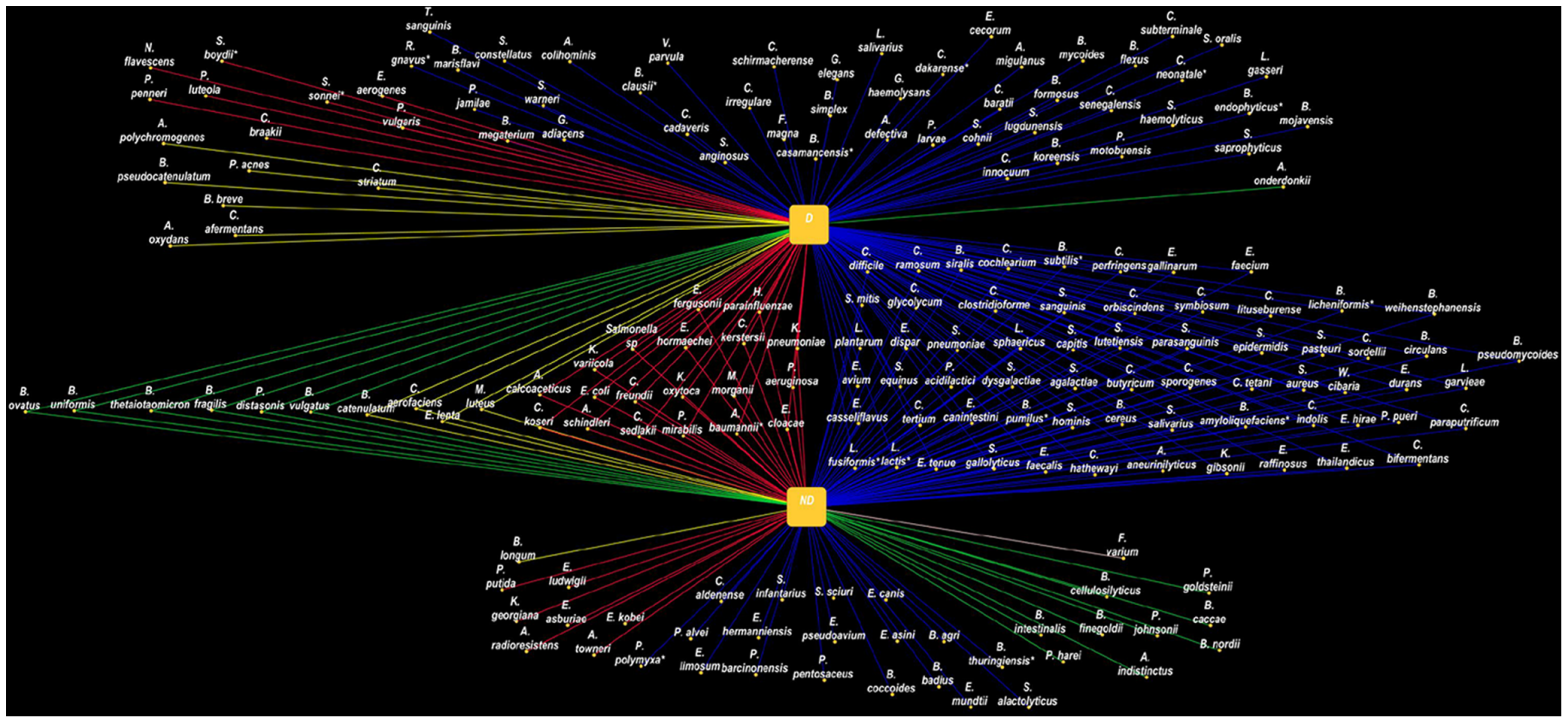
Isolates from individuals with diarrhea (D; top) and without diarrhea (ND; bottom). Each bacterial species corresponds to a node. The edge color represents the phylum (blue: *Firmicutes*; red: *Proteobacteria*; green: *Bacteroidetes*; yellow: *Actinobacteria*; pink: *Fusobacteria*). The common and specific bacteria detected from patients with diarrhea and those without are provided.

Overall, 2,753 isolates were tested, which allowed us to identify 189 bacterial species from 5 phyla, including an unknown species and 3 fungi (Table 4 and Figure 1) [19]. Two stool specimens from patients with diarrhea did not allow for the recovery of any bacteria. *Candida albicans* was detected from 3 patients with diarrhea (3/162 versus 0/185, *P* = 0.1). A total of 1,175 bacterial isolates were detected among patients with diarrhea and 1,575 were detected among patients without diarrhea. The number of different bacterial species per stool sample was significantly higher among patients without diarrhea (mean of 8.6±3, range 1 to 18) than among those with diarrhea (mean of 7.3±3.4, range 0 to 22; *P* = 0.0003). Finally, 59 out of the 153 bacterial species (38.6%) identified among patients with diarrhea were specific for this group whereas 36 out of the 129 bacterial species (27.9%) identified among patients without diarrhea were specific for this group, although this difference is not significant (*P* = 0.059).

**Table 4:** Comparison between the prevalence of 189 bacterial species identified among 2,750 isolates from fecal samples of 162 individuals with diarrhea and 185 without diarrhea.

		162 withdiarrhea		185 withoutdiarrhea		Total = 347		
Phyla	Bacteria	N° ofisolate	%	N° ofisolate	%	N° ofisolate	%	*P* *value*
**>100**								
***Proteobacteria***	*Escherichia coli*	**104**	64.2	**157**	84.9	261	75.2	**≤10** ^−**3**^
***Firmicutes***	*Enterococcus faecium*	**102**	63	**154**	83.2	256	73.8	**≤10** ^−**3**^
***Firmicutes***	*Clostridium bifermentans*	**60**	37	**99**	53.5	159	45.8	**0.002**
***Firmicutes***	*Enterococcus faecalis*	**76**	46.9	**77**	41.6	153	44	ns
***Firmicutes***	*Clostridium perfringens*	**53**	32.7	**99**	53.5	152	43.8	**≤10** ^−**3**^
***Firmicutes***	*Bacillus cereus*	**57**	35.2	**80**	43.2	137	39.5	ns
***Firmicutes***	*Enterococcus hirae*	**48**	29.7	**58**	31.3	106	30.5	ns
**>10–100**								
***Firmicutes***	*Enterococcus gallinarum*	**34**	21	**52**	28.1	86	24.8	ns
***Proteobacteria***	*Klebsiella pneumoniae*	**33**	20.4	**51**	27.6	84	24.2	ns
***Firmicutes***	*Clostridium sordellii*	**29**	17.9	**48**	25.9	77	22.2	ns
***Firmicutes***	*Lactococcus garvieae*	**23**	14.2	**34**	18.4	57	16.4	ns
***Bacteroidetes***	*Bacteroides fragilis*	**20**	12.5	**35**	18.9	55	15.8	ns
***Firmicutes***	*Enterococcus avium*	**20**	12.3	**32**	17.3	52	14.5	ns
***Firmicutes***	*Clostridium orbiscindens*	**12**	7.4	**30**	16.2	42	12.1	**0.01**
***Proteobacteria***	*Enterobacter cloacae*	**23**	14.2	**18**	9.7	41	11.8	ns
***Bacteroidetes***	*Bacteroides uniformis*	**8**	5	**30**	16.2	38	10.9	**0.001**
***Firmicutes***	*Bacillus subtilis* 1	**22**	13.6	**15**	8.1	37	10.7	ns
***Firmicutes***	*Clostridium symbiosum*	**10**	6.2	**25**	13.5	35	10	**0.03**
***Firmicutes***	*Enterococcus casseliflavus*	**10**	6.2	**25**	13.5	35	10	**0.03**
***Bacteroidetes***	*Bacteroides thetaiotaomicron*	**9**	5.5	**21**	11.3	30	8.6	ns
***Firmicutes***	*Streptococcus equinus*	**13**	8	**16**	8.6	29	8.4	ns
***Actinobacteria***	*Collinsella aerofaciens*	**6**	3.7	**20**	10.8	26	7.5	**0.01**
***Firmicutes***	*Bacillus pumilus* 1	**19**	11.7	**7**	3.8	26	7.5	**0.002**
***Firmicutes***	*Streptococcus lutetiensis*	**16**	9.9	**10**	5.4	26	7.5	ns
***Firmicutes***	*Lysinibacillus fusiformis* 1	**4**	2.5	**21**	11.3	25	7.2	**0.001**
***Bacteroidetes***	*Bacteroides ovatus*	**10**	6	**14**	7.6	24	6.9	ns
***Firmicutes***	*Streptococcus gallolyticus*	**16**	9.9	**8**	4.3	24	6.9	ns
***Proteobacteria***	*Proteus mirabilis*	**9**	5.6	**15**	8.1	24	6.9	ns
***Actinobacteria***	*Eggerthella lenta*	**4**	2.5	**19**	10.3	23	6.6	**0.004**
***Proteobacteria***	*Comamonas kerstersii*	**8**	4.9	**12**	6.5	20	5.8	ns
***Firmicutes***	*Clostridium butyricum*	**6**	3.7	**13**	7	19	5.5	ns
***Firmicutes***	*Clostridium glycolycum*	**5**	3	**14**	7.6	19	5.5	ns
***Bacteroidetes***	*Bacteroides vulgatus*	**2**	1.2	**16**	8.7	18	5.2	**≤10** ^−**3**^
***Firmicutes***	*Bacillus amyloliquefaciens* 1	**10**	6.2	**8**	4.3	18	5.2	ns
***Firmicutes***	*Clostridium tertium*	**11**	6.8	**7**	3.8	18	5.2	ns
***Firmicutes***	*Clostridium cochlearium*	**4**	2.5	**12**	6.5	16	4.6	ns
***Bacteroidetes***	*Parabacteroides distasonis*	**7**	4.3	**8**	4.3	15	4.3	ns
***Proteobacteria***	*Morganella morganii*	**6**	3.7	**9**	4.9	15	4.3	ns
***Firmicutes***	*Bacillus licheniformis* 1	**10**	6.2	**3**	1.6	13	3.7	**0.02**
***Firmicutes***	*Clostridium lituseburense* 1	**4**	2.5	**9**	4.9	13	3.7	ns
***Firmicutes***	*Kurthia gibsonii* 1	**2**	1.2	**11**	5.9	13	3.7	**0.02**
***Firmicutes***	*Clostridium ramosum*	**5**	3	**7**	3.8	12	3.5	ns
***Firmicutes***	*Staphylococcus aureus*	**10**	6.2	**2**	1	12	3.5	**0.01**
***Firmicutes***	*Weissella cibaria* 1	**4**	2.5	**8**	4.3	12	3.5	ns
***Proteobacteria***	*Acinetobacter baumannii* 1	**4**	2.5	**7**	3.8	11	4	ns
***Firmicutes***	*Streptococcus parasanguinis*	**8**	4.9	**3**	1.6	11	3.2	ns
**1–10 isolates**								
***Firmicutes***	*Bacillus circulans*	**5**	3	**5**	2.7	10	2.9	ns
***Firmicutes***	*Bacillus weihenstephanensis*	**5**	3	**4**	2.2	9	2.6	ns
***Firmicutes***	*Enterococcus thailandicus* 2	**3**	1.8	**6**	3.2	9	2.6	ns
***Firmicutes***	*Streptococcus pneumoniae*	**5**	3	**4**	2.2	9	2.6	ns
***Firmicutes***	*Enterococcus canintestini*	**4**	2.5	**4**	2.2	8	2.3	ns
***Firmicutes***	*Enterococcus durans*	**6**	3.7	**2**	1	8	2.3	ns
***Firmicutes***	*Staphylococcus epidermidis*	**7**	4.3	**1**	0.5	8	2.3	**0.02**
***Actinobacteria***	*Micrococcus luteus*	**5**	3	**2**	1	7	2	ns
***Firmicutes***	*Bacillus siralis*	**3**	1.8	**4**	2.2	7	2	ns
***Firmicutes***	*Enterococcus dispar*	**4**	2.5	**3**	1.6	7	2	ns
***Firmicutes***	*Enterococcus raffinosus* 3	**3**	1.8	**4**	2.2	7	2	ns
***Proteobacteria***	*Enterobacter hormaechei*	**5**	3	**2**	1	7	2	ns
***Firmicutes***	*Aneurinibacillus aneurinilyticus*	**2**	1.2	**4**	2.2	6	1.7	ns
***Firmicutes***	*Clostridium sporogenes*	**4**	2.5	**2**	1	6	1.7	ns
***Firmicutes***	*Streptococcus agalactiae*	**4**	2.5	**2**	1	6	1.7	ns
***Firmicutes***	*Streptococcus dysgalactiae* 3	**5**	3	**1**	0.5	6	1.7	ns
***Proteobacteria***	*Citrobacter freundii*	**1**	0.6	**5**	2.7	6	1.7	ns
***Firmicutes***	*Eubacterium limosum*	**0**	0	**5**	2.7	5	1.4	**0.04**
***Firmicutes***	*Paenibacillus pueri*	**2**	1.2	**3**	1.6	5	1.4	ns
***Firmicutes***	*Staphylococcus haemolyticus*	**5**	3	**0**	0	5	1.4	**0.02**
***Firmicutes***	*Streptococcus salivarius*	**4**	2.5	**1**	0.5	5	1.4	ns
***Proteobacteria***	*Acinetobacter calcoaceticus*	**2**	1.2	**3**	1.6	5	1.4	ns
***Proteobacteria***	*Escherichia fergusonii*	**3**	1.8	**2**	1	5	1.4	ns
***Proteobacteria***	*Klebsiella oxytoca*	**2**	1.2	**3**	1.6	5	1.4	ns
***Actinobacteria***	*Bifidobacterium breve*	**4**	2.5	**0**	0	4	1.1	**0.047**
***Actinobacteria***	*Propionibacterium acnes*	**4**	2.5	**0**	0	4	1.1	**0.047**
***Firmicutes***	*Bacillus mojavensis*	**4**	2.5	**0**	0	4	1.1	**0.04**7
***Firmicutes***	*Clostridium clostridioforme* 1	**3**	1.8	**1**	0.5	4	1.1	ns
***Firmicutes***	*Clostridium hathewayi*	**3**	1.8	**1**	0.5	4	1.1	ns
***Firmicutes***	*Clostridium paraputrificum*	**3**	1.8	**1**	0.5	4	1.1	ns
***Firmicutes***	*Enterococcus asini*	**0**	0	**4**	2.2	4	1.1	ns
***Firmicutes***	*Finegoldia magna*	**4**	2.5	**0**	0	4	1.1	**0.047**
***Firmicutes***	*Lactococcus lactis* 1	**1**	0.6	**3**	1.6	4	1.1	ns
***Firmicutes***	*Streptococcus anginosus*	**4**	2.5	**0**	0	4	1.1	**0.047**
***Proteobacteria***	*Enterobacter asburiae*	**0**	0	**4**	2.2	4	1.1	ns
***Proteobacteria***	*Haemophilus parainfluenzae*	**3**	1.8	**1**	0.5	4	1.1	ns
***Proteobacteria***	*Pseudomonas aeruginosa*	**2**	1.2	**2**	1	4	1.1	ns
***Proteobacteria***	*Salmonella enterica*	**3**	1.8	**1**	0.5	4	1.1	ns
***Firmicutes***	*Lactobacillus gasseri*	**3**	1.8	**0**	0	3	0.9	ns
***Firmicutes***	*Lactobacillus plantarum*	**1**	0.6	**2**	1	3	0.9	ns
***Firmicutes***	*Paenibacillus jamilae* 2	**3**	1.8	**0**	0	3	0.9	ns
***Firmicutes***	*Paenibacillus larvae* 3	**3**	1.8	**0**	0	3	0.9	ns
***Firmicutes***	*Staphylococcus capitis*	**2**	1.2	**1**	0.5	3	0.9	ns
***Firmicutes***	*Staphylococcus hominis*	**2**	1.2	**1**	0.5	3	0.9	ns
***Firmicutes***	*Staphylococcus lugdunensis*	**3**	1.8	**0**	0	3	0.9	ns
***Firmicutes***	*Staphylococcus pasteuri*	**2**	1.2	**1**	0.5	3	0.9	ns
***Firmicutes***	*Streptococcus alactolyticus* 3	**0**	0	**3**	1.6	3	0.9	ns
***Proteobacteria***	*Enterobacter kobei*	**0**	0	**3**	1.6	3	0.9	ns
***Proteobacteria***	*Acinetobacter schindleri*	**1**	0.6	**1**	0.5	2	0.6	ns
***Actinobacteria***	*Bifidobacterium catenulatum*	**1**	0.6	**1**	0.5	2	0.6	ns
***Actinobacteria***	*Bifidobacterium longum*	**0**	0	**2**	1	2	0.6	ns
***Bacteroidetes***	*Parabacteroides goldsteinii* 1	**0**	0	**2**	1	2	0.6	ns
***Bacteroidetes***	*Parabacteroides johnsonii*	**0**	0	**2**	1	2	0.6	ns
***Firmicutes***	*Aneurinibacillus migulanus* 1	**2**	1.2	**0**	0	2	0.6	ns
***Firmicutes***	*Bacillus badius*	**0**	0	**2**	1	2	0.6	ns
***Firmicutes***	*Bacillus endophyticus* 1	**2**	1.2	**0**	0	2	0.6	ns
***Firmicutes***	*Bacillus megaterium*	**2**	1.2	**0**	0	2	0.6	ns
***Firmicutes***	*Bacillus pseudomycoides* 2	**1**	0.6	**1**	0.5	2	0.6	ns
***Firmicutes***	*Clostridium aldenense*	**0**	0	**2**	1	2	0.6	ns
***Firmicutes***	*Clostridium difficile*	**1**	0.6	**1**	0.5	2	0.6	ns
***Firmicutes***	*Clostridium indolis*	**1**	0.6	**1**	0.5	2	0.6	ns
***Firmicutes***	*Clostridium innocuum*	**2**	1.2	**0**	0	2	0.6	ns
***Firmicutes***	*Clostridium subterminale* 3	**2**	1.2	**0**	0	2	0.6	ns
***Firmicutes***	*Clostridium tetani* 3	**1**	0.6	**1**	0.5	2	0.6	ns
***Firmicutes***	*Enterococcus canis* 2	**0**	0	**2**	1	2	0.6	ns
***Firmicutes***	*Enterococcus cecorum*	**2**	1.2	**0**	0	2	0.6	ns
***Firmicutes***	*Enterococcus pseudoavium* 3	**0**	0	**2**	1	2	0.6	ns
***Firmicutes***	*Enterococcus tenue*	**1**	0.6	**1**	0.5	2	0.6	ns
***Firmicutes***	*Lysinibacillus sphaericus* 1	**1**	0.6	**1**	0.5	2	0.6	ns
***Firmicutes***	*Paenibacillus alvei*	**0**	0	**2**	1	2	0.6	ns
***Firmicutes***	*Pediococcu acidilactici*	**1**	0.6	**1**	0.5	2	0.6	ns
***Firmicutes***	*Ruminocus gnavus* 1	**2**	1.2	**0**	0	2	0.6	ns
***Firmicutes***	*Streptococcus infantarius*	**0**	0	**2**	1	2	0.6	ns
***Firmicutes***	*Streptococcus mitis*	**1**	0.6	**1**	0.5	2	0.6	ns
***Firmicutes***	*Streptococcus oralis*	**2**	1.2	**0**	0	2	0.6	ns
***Firmicutes***	*Streptococcus sanguinis*	**1**	0.6	**1**	0.5	2	0.6	ns
***Proteobacteria***	*Acinetobacter radioresistens*	**0**	0	**2**	1	2	0.6	ns
***Proteobacteria***	*Citrobacter koseri*	**1**	0.6	**1**	0.5	2	0.6	ns
***Proteobacteria***	*Citrobacter sedlakii*	**1**	0.6	**1**	0.5	2	0.6	ns
***Proteobacteria***	*Klebsiella variicola*	**1**	0.6	**1**	0.5	2	0.6	ns
***Proteobacteria***	*Proteus vulgaris*	**2**	1.2	**0**	0	2	0.6	ns
**1 isolate**								
***Actinobacteria***	*Arthrobacter polychromogenes*	**1**	0.6	**0**	0	1	0.3	ns
***Actinobacteria***	*Arthrobacter oxydans*	**1**	0.6	**0**	0	1	0.3	ns
***Actinobacteria***	*Bifidobacterium pseudocatenulatum*	**1**	0.6	**0**	0	1	0.3	ns
***Actinobacteria***	*Corynebacterium afermentans*	**1**	0.6	**0**	0	1	0.3	ns
***Actinobacteria***	*Corynebacterium striatum*	**1**	0.6	**0**	0	1	0.3	ns
***Bacteroidetes***	*Alistipes indistinctus*	**0**	0	**1**	0.5	1	0.3	ns
***Bacteroidetes***	*Alistipes onderdonkii*	**1**	0.6	**0**	0	1	0.3	ns
***Bacteroidetes***	*Bacteroides caccae*	**0**	0	**1**	0.5	1	0.3	ns
***Bacteroidetes***	*Bacteroides cellulosilyticus*	**0**	0	**1**	0.5	1	0.3	ns
***Bacteroidetes***	*Bacteroides finegoldii*	**0**	0	**1**	0.5	1	0.3	ns
***Bacteroidetes***	*Bacteroides intestinalis*	**0**	0	**1**	0.5	1	0.3	ns
***Bacteroidetes***	*Bacteroides nordii* 1	**0**	0	**1**	0.5	1	0.3	ns
***Bacteroidetes***	*Peptoniphilus harei*	**0**	0	**1**	0.5	1	0.3	ns
***Firmicutes***	*Abiotrophia defectiva*	**1**	0.6	**0**	0	1	0.3	ns
***Firmicutes***	*Anaerotruncus colihominis*	**1**	0.6	**0**	0	1	0.3	ns
***Firmicutes***	*Bacillus casamancensis* 1 ^,^ 4	**1**	0.6	**0**	0	1	0.3	ns
***Firmicutes***	*Bacillus clausii* 1	**1**	0.6	**0**	0	1	0.3	ns
***Firmicutes***	*Bacillus flexus*	**1**	0.6	**0**	0	1	0.3	ns
***Firmicutes***	*Bacillus koreensis* 2	**1**	0.6	**0**	0	1	0.3	ns
***Firmicutes***	*Bacillus marisflavi*	**1**	0.6	**0**	0	1	0.3	ns
***Firmicutes***	*Bacillus mycoides*	**1**	0.6	**0**	0	1	0.3	ns
***Firmicutes***	*Bacillus simplex*	**1**	0.6	**0**	0	1	0.3	ns
***Firmicutes***	*Bacillus thuringiensis* 1	**0**	0	**1**	0.5	1	0.3	ns
***Firmicutes***	*Bacillus coccoides*	**0**	0	**1**	0.5	1	0.3	ns
***Firmicutes***	*Bacillus agri*	**0**	0	**1**	0.5	1	0.3	ns
***Firmicutes***	*Bacillus formosus* 2	**1**	0.6	**0**	0	1	0.3	ns
***Firmicutes***	*Clostridium baratii*	**1**	0.6	**0**	0	1	0.3	ns
***Firmicutes***	*Clostridium cadaveris* 1	**1**	0.6	**0**	0	1	0.3	ns
***Firmicutes***	*Clostridium dakarense* 1 ^,^ 4	**1**	0.6	**0**	0	1	0.3	ns
***Firmicutes***	*Clostridium irregulare* 3	**1**	0.6	**0**	0	1	0.3	ns
***Firmicutes***	*Clostridium neonatale* 1	**1**	0.6	**0**	0	1	0.3	ns
***Firmicutes***	*Clostridium schirmacherense* 2	**1**	0.6	**0**	0	1	0.3	ns
***Firmicutes***	*Clostridium senegalense*	**1**	0.6	**0**	0	1	0.3	ns
***Firmicutes***	*Enterococcus hermanniensis* 2	**0**	0	**1**	0.5	1	0.3	ns
***Firmicutes***	*Enterococcus mundtii*	**0**	0	**1**	0.5	1	0.3	ns
***Firmicutes***	*Gemella haemolysans*	**1**	0.6	**0**	0	1	0.3	ns
***Firmicutes***	*Granulicatella adiacens*	**1**	0.6	**0**	0	1	0.3	ns
***Firmicutes***	*Granulicatella elegans*	**1**	0.6	**0**	0	1	0.3	ns
***Firmicutes***	*Lactobacillus salivarius*	**1**	0.6	**0**	0	1	0.3	ns
***Firmicutes***	*Paenibacillus barcinonensis*	**0**	0	**1**	0.5	1	0.3	ns
***Firmicutes***	*Paenibacillus motobuensis* 2	**1**	0.6	**0**	0	1	0.3	ns
***Firmicutes***	*Paenibacillus polymyxa* 1 ^,^ 3	**0**	0	**1**	0.5	1	0.3	ns
***Firmicutes***	*Pediococcus pentosaceus*	**0**	0	**1**	0.5	1	0.3	ns
***Firmicutes***	*Staphylococcus cohnii*	**1**	0.6	**0**	0	1	0.3	ns
***Firmicutes***	*Staphylococcus saprophyticus*	**1**	0.6	**0**	0	1	0.3	ns
***Firmicutes***	*Staphylococcus sciuri* 1	**0**	0	**1**	0.5	1	0.3	ns
***Firmicutes***	*Staphylococcus warneri*	**1**	0.6	**0**	0	1	0.3	ns
***Firmicutes***	*Streptococcus constellatus*	**1**	0.6	**0**	0	1	0.3	ns
***Firmicutes***	*Turicibacter sanguinis*	**1**	0.6	**0**	0	1	0.3	ns
***Firmicutes***	*Veillonella parvula*	**1**	0.6	**0**	0	1	0.3	ns
***Fusobacteria***	*Fusobacterium varium*	**0**	0	**1**	0.5	1	0.3	ns
***Proteobacteria***	*Acinetobacter towneri* 2	**0**	0	**1**	0.5	1	0.3	ns
***Proteobacteria***	*Citrobacter braakii*	**1**	0.6	**0**	0	1	0.3	ns
***Proteobacteria***	*Enterobacter aerogenes*	**1**	0.6	**0**	0	1	0.3	ns
***Proteobacteria***	*Enterobacter ludwigii* 3	**0**	0	**1**	0.5	1	0.3	ns
***Proteobacteria***	*Kluyvera georgiana* 3	**0**	0	**1**	0.5	1	0.3	ns
***Proteobacteria***	*Neisseria flavescens*	**1**	0.6	**0**	0	1	0.3	ns
***Proteobacteria***	*Proteus penneri*	**1**	0.6	**0**	0	1	0.3	ns
***Proteobacteria***	*Pseudomonas luteola*	**1**	0.6	**0**	0	1	0.3	ns
***Proteobacteria***	*Pseudomonas putida*	**0**	0	**1**	0.5	1	0.3	ns
***Proteobacteria***	*Shigella boydii* 1	**1**	0.6	**0**	0	1	0.3	ns
***Proteobacteria***	*Shigella sonnei* 1	**1**	0.6	**0**	0	1	0.3	ns

P value is specified only when a significant difference was observed.

N° of isolate*:* Number of isolate*; %: Percentage;* ns: non significant value.

1 Strains identified using a molecular analysis;

2 Bacterial species that were never isolated in humans;

3 Bacterial species isolated in humans but not in the human gut;

4 New bacterial species.

### MALDI-TOF Mass Spectrometry Identification

Of the 2,750 bacterial isolates analyzed, 2,718 (98.8%) yielded an accurate identification using MALDI-TOF mass spectrometry (Table 4).

### 16S rRNA Amplification and Sequencing Identification

Thirty-two isolates out of the 2,750 (1.2%) were not identified by MALDI-TOF mass spectrometry. Among these isolates, 11 were identified using 16S rRNA sequencing: *Bacteroides nordii*, *Bacillus clausii, Bacillus thuringiensis*, *Clostridium cadaveris*, *Clostridium neonatale*, *Paenibacillus polymyxa*, *Staphylococcus sciuri, Shigella boydii*, *Shigella sonnei*, and two new species were identified: a new clostridial species that was called *Clostridium dakarense* sp. nov. (GenBank accession number KC517358) and a new *Bacillus* species, *Bacillus casamencensis* sp. nov. (GenBank accession number AF519462.1). The 16S rRNA sequence of this *Bacillus* species has been already detected in rice soils in Senegal but no description of the bacterium has been yet reported. The full genome of *C. dakarense* has been recently sequenced and reported [20].

The other isolates identified by 16S rRNA sequence included 1 of 2 *Parabacteroides goldsteinii* isolates detected in the study, 1 of 2 *Aneurinibacillus migulanus* isolates, 2 (11%) of 18 *Bacillus amyloliquefaciens* isolates, 1 of 2 *Bacillus endophyticus* isolates, 1 (7.7%) of 13 *Bacillus licheniformis* isolates, 2 (7.7%) of 26 *Bacillus pumilus* isolates, 3 (8%) of 37 *Bacillus subtilis*, 1 of 4 *Clostridium clostridioforme* isolates, 1 of 13 (7.7%) *Clostridium lituseburense* isolates, 1 of 13 (7.7%) *Kurthia gibsonii* isolates, 1 of 4 *Lactococcus lactis* isolates, 1 of 25 (4%) *Lysinibacillus fusiformis* isolates, 1 of 2 *Lysinibacillus sphaericus* isolates, 1 of 2 *Ruminococcus gnavus* isolates, 1 of 12 (8.3%) *Weissella cibaria* isolates, and 2 of 11 (18.2%) *Acinetobacter baumannii* isolates. When the spectra of the aforementioned isolates were added to the Bruker database, further identifications of these organisms by MALDI-TOF were accurate.

#### Common bacteria

**Figure 2:**
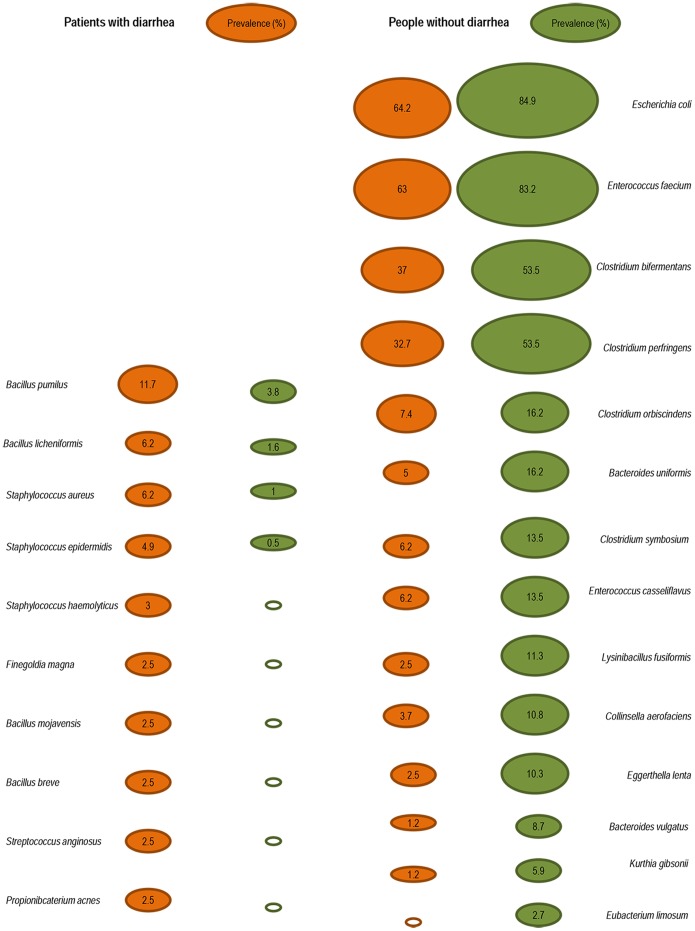
Bacterial species for which significant differences have been observed between individuals with diarrhea and those without diarrhea.

Seven bacterial species (3.7%) were identified more than 100 times in fecal samples (261 *Escherichia coli* isolates, 256 *Enterococcus faecium* isolates, 159 *Clostridium bifermentans* isolates, 153 *Enterococcus faecalis* isolates, 152 *Clostridium perfringens* isolates, 137 *Bacillus cereus* isolates, and 106 *Enterococcus hirae* isolates). Surprisingly, several bacteria were more common in patients without diarrhea including *E. coli* than those without (*P≤*10^−3^), *E. faecium* (*P≤*10^−3^), *C. bifermentans* (*P* = 0.002), and *C. perfringens* (*P≤*10^−3^), see Table 4 and Figure 2.

Thirty-nine bacterial species (20.6%) from 18 different genera were identified from between 10 and 100 fecal samples (Table 4 and Figure 2). Several were more common in patients with diarrhea than those without, such as *Bacillus licheniformis* (*P* = 0.02), *Bacillus pumilus* (*P* = 0.002), and *Staphylococcus aureus* (*P* = 0.01). In contrast, people without diarrhea had more commonly *Lysinibacillus fusiformis* (*P = *0.001), *Clostridium orbiscindens* (*P* = 0.01), *Clostridium symbiosum* (*P* = 0.03), *Enterococcus casseliflavus* (*P* = 0.03), *Kurthia gibsonii* (*P* = 0.02), and *Collinsella aerofaciens* (*P* = 0.01), *Eggerthella lenta* (*P* = 0.004), *Bacteroides uniformis* (*P = *0.001), and *Bacteroides vulgatus* (*P = *0.03).

#### Rare bacterial species

Overall, 81 out of 189 bacterial species (43%) were identified from between 2 and 10 fecal samples (Table 4). Among them, *Bifidobacterium breve, Propionibacterium acnes*, *Bacillus mojavensis*, *Finegoldia magna*, and *Streptococcus anginosus* were each detected in only 4 patients with diarrhea (*P* = 0.047). *Staphylococcus haemolyticus* was detected in only 5 patients with diarrhea (*P = *0.02). *Staphylococcus epidermidis* was significantly more frequent among people with diarrhea (7/162) than among those without (1/185, *P* = 0.02). In contrast, *Eubacterium limosum* was identified only in 5 people without diarrhea (*P* = 0.04).

#### Bacterial species isolated only once

Overall, 51 bacterial species were identified only once (Table 4). Among them, 5 different bacterial species from the phylum *Actinobacteria*, 5 from the genera *Bacillus,* 4 from the genera *Clostridium*, and 2 from the genera *Shigella* were detected among patients with diarrhea. In contrast, several species of the genera *Bacteroides* (4) and *Enterococcus* (2) were detected only among patients without diarrhea.

#### Bacterial identification depending of the age range

The isolates obtained from people with and without diarrhea depending of the age range (less than 5 years, from 5 to 20 years, and more than 20 years) were compared. Only significant differences are presented (Table S1). For children from 0 to 5 year-old, 2 species of the genera *Clostridium* were significantly more frequent among those without diarrhea, including 1 species *C. glycolycum*, for which the data were not significant when the entire population was analyzed. For adult of more than 20 year-old, 6 species (*E. coli*, *E. faecium*, *B. uniformis*, *B. vulgatus*, *C. orbiscindens*, and *E. lenta*), as previously observed in the entire population, were significantly more observed in people without diarrhea. In contrast, those with diarrhea had more commonly *S. aureus*, *F. magna*, *B. pumilus*, as previously observed, as well as another *Bacillus* species, *B. subtilis.* For people from 5 to 20 year-old, *E. faecium*, *C. perfringens*, and *C. symbosium* were significantly more detected in people without diarrhea, as observed in the entire population. Finally, the comparison of the isolates from people with diarrhea between them depending of the age range did not yield statistically significant results.

### Viral and Parasites Identification

Analyses in Dakar have allowed the detection of several viruses and parasites in feces. Ten rotaviruses (6.2%), 4 adenoviruses (2.7%), and 7 co-infections with both rotaviruses and adenoviruses (4.3%) were detected among diarrheic patients. Sixteen *Enterobius vermicularis* (9.9%), 6 *Trichomonas intestinalis* (3.7%), 5 *Cryptosporidium* spp. (3%), 5 cysts of *Entamoeba* spp. (3%), 4 *Schistosoma mansoni* (2.7%), and 1 *Microsporidium* spp. (0.6%) were detected among 37 diarrheic people. Thirty-six *Ascaris lumbricoides* (among 24 diarrheic people and 12 without diarrhea), 8 *Giardia duodenalis* (among 6 diarrheic people and 2 without diarrhea), and 4 *Trichuris trichiura* (among 1 diarrheic people and 3 without) were detected. Finally, 2 co-infections (*Cryptosporidium* spp. with *Ascaris lumbricoides* and *Microsporidium* spp. with *Ascaris lumbricoides*) were detected in patients with diarrhea and 1 co-infection (*Trichuris trichiura* with *Ascaris lumbricoides*) among a people without diarrhea.

## Discussion

MALDI-TOF mass spectrometry coupled with culturomics has allowed for the identification of a large collection of bacterial species from specimens from Senegal and a preliminary comparison between the bacterial microbiota of people with and without diarrhea. This technique has allowed for the accurate identification of a large panel of anaerobes that are usually poorly identified by current phenotypic methods, which lack specificity and result in ambiguous or even erroneous identification [21], [22]. For several bacterial species, their identification by MALDI-TOF failed because either the corresponding species missed in the database or either the number of spectra of the species was insufficient. Indeed, the continuous increases of the entries in database with the addition of our new spectra solved these problems and improved bacterial identification. In addition, the use of MALDI-TOF mass spectrometry detects the presence of previously rare bacteria that were difficult to identify using phenotypic methods [6], [23]–[27].

Overall, the percentage of isolates from Senegal that were correctly identified at the genus and species level by mass spectrometry (98.2%) is nearly the same than the percentage (95.4%) observed in the first large scale experiment that used mass spectrometry in Marseille, France [1]. Both studies were performed using the same database. This study has allowed us to test a large collection of isolated strains from Senegalese people. Only 3 bacterial species, *Clostridium senegalense*, *Bacillus casamancensis*, and *Clostridium dakarense*, have been currently identified in Senegal. This confirms the high potential for culturomics approaches to result in the detection of new bacterial species associated with humans [28]–[39]. The increases in the database by the addition of more bacteria have allowed for improved bacterial identification by MALDI-TOF mass spectrometry. Thus, the current database seems accurate for the identification of bacteria in Senegal. This work allowed for the identification of 166 bacterial species already found in the human gut, 11 species previously detected in humans but not in the gut, 10 species detected in humans for the first time, and 2 unknown species.

The composition of the gut microbiota is complex [40]. A recent culturomics experiment using many culture conditions was performed on fecal samples from 2 healthy Senegalese individuals, 1 obese person, 1 person with resistant tuberculosis, and a patient with anorexia nervosa. This allowed the identification of 99, 219, 192, 39, and 133 different bacterial species per fecal sample, respectively [12]–[14]. Although the storage and transport conditions of the fecal samples were not optimal and many fewer culture conditions were used, this study demonstrates a modification of gut microbiota with several significant differences between the bacterial species identified among people with diarrhea and those without diarrhea. In people with diarrhea, major commensal bacterial species such as *E. coli* were significantly decreased, as were several *Enterococcus* spp. (*E. faecium* and *E. casseliflavus*); anaerobes, such as *Bacteroides* spp. (*B. uniformis* and *B. vulgatus*); and *Clostridium* spp. (*C. bifermentans*, *C. orbiscindens*, *C. perfringens*, *C. symbosium,* and *C. glycolycum*). Conversely, several *Bacillus* spp. (*B. licheniformis*, *B. mojavensis*, *B. pumilus*, and *B. subtilis*) were significantly more frequent among patients with diarrhea. In addition, the diversity of *Bacillus* species identified in patients with diarrhea is higher (19) than among those without diarrhea (11), but this difference was not significant (*P* = 0.055). Overall, a decrease of anaerobes in the gut flora, particularly Bacteroidetes, has already been reported during gastroenteritis using both culture and molecular methods [41], [42]. Our data shows the occurrence of an imbalance of natural bacterial flora among patients with diarrhea.

For a long time, the high cost of a MALDI-TOF apparatus and the lack of specific reagent have limited the development of this technology. The expense of using MALDI-TOF mass spectrometry for identification now lies in the acquisition of a machine, which costs between €100,000 and €200,000 [21]. Recently, the cost per sample was calculated to be 1.35 euros for the Microflex system from Bruker [21]. The time required for bacterial identification has been improved to 1 minute 46 seconds using the Microflex system. In addition, MALDI-TOF mass spectrometry also has the potential for identification at the serotype level and antibiotic resistance profiling within minutes [43]–[51]. Thus, the rapid and accurate identification of routinely encountered bacterial species can be performed to improve the care of patients with infectious diseases. This technique will be a promising alternative for bacterial identification in Africa. Indeed, the main cost is based on the investment of purchasing the apparatus. The used reagents do not expire, do not require specific storage conditions, and are not expensive [1], [6]. Finally, the protocol that involves directly deposited bacterial colonies onto the MALDI-TOF mass spectrometry plate regardless of the agar-based medium and without any subculture or colony preparation is very simple and can be widely used.

Overall, MALDI-TOF mass spectrometry is a potentially powerful tool for routine bacterial identification in Africa, as it allows for the rapid identification of bacterial species, including those that are rare and difficult to identify using phenotypic methods. The next step will be to install MALDI-TOF mass spectrometers in African hospitals.

## Supporting Information

Table S1 **Summary of the significant differences observed between the prevalence of bacterial species from fecal samples of 347 individuals with and without diarrhea depending of the age range.**
(DOCX)
