# Supplementary material for: MALDI-TOF Identification of the Human Gut Microbiome in People with and without Diarrhea in Senegal
Source: PLoS One. 2014 May 1;9(5):e87419. doi: 10.1371/journal.pone.0087419 (PMC4006720; doi:10.1371/journal.pone.0087419)
Supplement: Table S1 — Summary of the significant differences observed between the prevalence of bacterial species from fecal samples of 347 individuals with and without diarrhea depending of the age range. (DOCX) [file pone.0087419.s001.docx]

**Table S1.** Summary of the significant differences observed between the prevalence of bacterial species from fecal samples of 347 individuals with and without diarrhea depending of the age range.

**a. 0-5 year-old**

|  |  | **71 with diarrhea** | | **9 without diarrhea** | | **Total** |  |  |
| --- | --- | --- | --- | --- | --- | --- | --- | --- |
| **Phyla** | **Bacteria** | **N° of isolate** | **%** | **N° of isolate** | **%** | **N° of isolate** | **%** | ***P value*** |
| ***Firmicutes*** | *Clostridium orbiscindens^1^* | **7** | 9.8 | **3** | 33.3 | 10 | 12.5 | **0.04** |
| ***Firmicutes*** | *Clostridium glycolycum^2^* | **2** | 2.8 | **2** | 22 | 4 | 5 | **0.03** |

**b. 5-20 year-old**

|  |  | **35 with diarrhea** | | **46 without diarrhea** | | **Total** |  |  |
| --- | --- | --- | --- | --- | --- | --- | --- | --- |
| **Phyla** | **Bacteria** | **N° of isolate** | **%** | **N° of isolate** | **%** | **N° of isolate** | **%** | ***P value*** |
| ***Firmicutes*** | *Enterococcus faecium^1^* | **23** | 65.7 | **39** | 84.8 | 62 | 76.5 | **0.04** |
| ***Firmicutes*** | *Clostridium perfringens^1^* | **13** | 37.1 | **30** | 65.2 | 43 | 53 | **0.01** |
| ***Firmicutes*** | *Clostridium symbiosum^1^* | **1** | 2.9 | **9** | 19.6 | 10 | 12.4 | **0.02** |

**C. More than 20 year-old**

|  |  | **56 with diarrhea** | | **130 without diarrhea** | | **Total** |  |  |
| --- | --- | --- | --- | --- | --- | --- | --- | --- |
| **Phyla** | **Bacteria** | **N° of isolate** | **%** | **N° of isolate** | **%** | **N° of isolate** | **%** | ***P value*** |
| ***Proteobacteria*** | *Escherichia coli^1^* | **39** | 69.6 | **115** | 88.5 | 154 | 82.8 | **0.001** |
| ***Firmicutes*** | *Enterococcus faecium^1^* | **37** | 66 | **109** | 83.8 | 146 | 78.5 | **0.006** |
| ***Bacteroidetes*** | *Bacteroides uniformis^1^* | **3** | 5.3 | **26** | 20 | 29 | 15.6 | **0.01** |
| ***Firmicutes*** | *Clostridium orbiscindens^1^* | **2** | 3.6 | **23** | 17.7 | 25 | 13.4 | **0.009** |
| ***Bacteroidetes*** | *Bacteroides vulgatus^1^* | **1** | 1.8 | **13** | 10 | 14 | 7.5 | **0.04** |
| ***Actinobacteria*** | *Eggerthella lenta^1^* | **0** | 0 | **13** | 10 | 13 | 7 | **0.007** |
| ***Firmicutes*** | *Bacillus subtilis^2^* | **8** | 14.3 | **7** | 5.4 | 15 | 8 | **0.04** |
| ***Firmicutes*** | *Bacillus pumilus^1^* | **7** | 12.5 | **3** | 2.4 | 10 | 5.4 | **0.008** |
| ***Firmicutes*** | *Staphylococcus aureus^1^* | **4** | 7 | **1** | 0.7 | 5 | 2.7 | **0.02** |
| ***Firmicutes*** | *Finegoldia magna^1^* | **3** | 5.4 | **0** | 0 | 3 | 1.6 | **0.02** |

^1^The difference is also significant when the entire population is compared.

^2^The difference is not significant when the entire population was compared.
